# Chromosome-Level Genome Assembly of the Rare and Endangered Tropical Plant *Speranskia yunnanensis* (Euphorbiaceae)

**DOI:** 10.3389/fgene.2021.755564

**Published:** 2022-01-24

**Authors:** Guofang Yuan, Shufang Tan, Dandan Wang, Yongzhi Yang, Bin Tian

**Affiliations:** ^1^ Key Laboratory for Forest Resources Conservation and Utilization in the Southwest Mountains of China, Ministry of Education, Southwest Forestry University, Kunming, China; ^2^ State Key Laboratory of Grassland Agro- Ecosystems, Institute of Innovation Ecology and School of Life Sciences, Lanzhou University, Lanzhou, China; ^3^ CAS Key Laboratory for Plant Diversity and Biogeography, Kunming Institute of Botany, Chinese Academy of Sciences, Kunming, China

**Keywords:** genome assembly, chromosome-level genome, phylogenetic relationships, evolution, *Speranskia yunnanensis*

*Speranskia yunnanensis* S. M. Hwang is an endangered shrub narrowly distributed in tropical regions, and its populations are gradually shrinking. We assembled and annotated the genome of *S. yunnanensis* at the chromosome level by combining Nanopore sequencing, Illumina HiSeq sequencing and Hi-C technology. The final genome assembly was ~417.65 Mb, with a contig N50 value of 12.52 Mb, and 408.62 Mb (97.84%) of which could be grouped into seven pseudochromosomes. Approximately 69.11% of the assembly was identified as repetitive elements, and 25,467 protein-coding genes were annotated. Based on the 1,517 single-copy orthologous genes, and 751 expanded and 1,645 contracted gene families among the 16,389 gene families in *S. yunnanensis*, a phylogenetic tree was further built. The high-quality, annotated, and chromosome-level genome of *S. yunnanensis* will present an important source of data for future research on the evolution of Euphorbiaceae genomes, and provide genomic resources toward studies on speciation, local adaptation, as well as conservation genomics of the ecologically important genus *Speranskia*.

## Introduction

*Speranskia* Baill., a small genus within the tribe Ricineae subfamily Acalyphoideae of Euphorbiaceae, is endemic in China (Hwang, 1989; Webster, 2014). Three members are recognized in *Speranskia*, namely *S. cantonensis* (Hance) Pax et Hoffm, *S. tuberculata* (Bunge) Baill., and *S. yunnanensis* Hwang. Of them, *S. cantonensis* and *S. tuberculata* are widely distributed from southwestern to northern China, whereas *S. yunnanensis* maintains a narrow distribution in tropical Yunnan and is found only in three small and fragmented natural populations (Hwang, 1989). As a member of the castor tribe (Ricineae) and a sister taxon to the castor bean (*Ricinus*) (Webster, 2014), *Speranskia* is important for us to infer the origin and evolution of castor bean as well as the evolution of ricin proteins. However, its genome composition and phylogenetic position within Ricineae are largely unknown. Here, we conducted a series of genomic analyses on *S. yunnanensis* (2n = 14), including chromosome-level assembly, annotations, phylogenetic reconstructions, gene family expansion and contraction analyses, divergence time estimation, and *Ks* analysis. The genome assembly and resources produced in this study will provide important insights as well as resource for future study in *S. yunnanensis* to facilitate conservation and in the genus *Speranskia* in general.

## Results and Discussion

### Library Preparation and Whole-Genome Sequencing

We sequenced the genome of *S. yunnanensis* S. M. Hwang using a combination of Illumina short-read sequencing, Oxford Nanopore Technologies, and Hi-C sequencing technologies. The short-insert library of 400 bp was constructed, and a total of 20.57 Gb of raw data was generated using HiSeq X ten of the Illumina platform. Low-quality reads were removed, and duplication reads were filtered to obtain 18.73 Gb of clean data (91.05%). Additionally, we obtained 31.35 Gb of pass reads from one flow cell of the PromethION sequencer for genome assembly. Furthermore, the Hi-C library was constructed and sequenced with paired-end (PE) 150 bp reads for chromosome-level scaffolding. Finally, approximately 65.16 Gb of Hi-C data was generated.

### Estimating *S. yunnanensis* Genome Size

Before genome assembly, a genome survey was performed to assess the genome size based on 18.73 Gb of Illumina clean data. Using a 21-mer analysis method, the major peak was located around a k-mer depth of 25, and the other clear peak was located at half of the expected depth inferred to be a heterozygous peak. The final predicted genome size of *S. yunnanensis* was ∼452.54 Mb with a heterozygosity rate of ∼0.62% and a repeat ratio of 53.4% (Supplementary Figure S1).

### De Novo Genome Assembly and Pseudochromosome Construction

Following self-error correction, Nanopore long reads were initially assembled into contigs with NextDenovo, which produced a preliminary assembly length of 414.61 Mb and a contig N50 size of 12.42 Mb (Supplementary Table S1). To further optimize the assembly, the preliminary assembly was polished using NextPolish. The final assembly of 417.65 Mb (92.29% of the predicted genome size) was obtained with a contig N50 size of 12.52 Mb (Supplementary Table S1). Based on 65.16 Gb of Hi-C data (∼163 × coverage), we obtained seven pseudochromosomes using JUICER and 3D-DNA programs. In total, 97.85% (408.62 Mb) of the assembly was anchored and oriented on pseudochromosomes with a scaffold N50 of 61.04 Mb, ranging from 45.31 to 68.49 Mb in length (Supplementary Figure S2 and Supplementary Table S2).

To evaluate our assembly, we first used the Benchmarking Universal Single-Copy Orthologs (BUSCO) to assess assembly completeness, and 98.3% Embryophyta-conserved genes could be completely predicted in our assembly (Supplementary Table S3). We then estimated the base accuracy of the assembly by mapping Illumina reads. In total, 94.24% of Illumina data could be mapped to the genome and 94.01 and 91.33% of the assembled genome sequence could be covered by at least 4- and 10-fold, respectively (Supplementary Table S4). Furthermore, GC content Poisson distributions presented a complete and high-quality genome assembly (Supplementary Figure S3).

### Repeat Annotation of the Genome Assembly

A total of ∼288.65 Mb repetitive elements (accounting for approximately 69.11% of the genome) were identified via two methods on the basis of *de novo* and homology-based predictions. Among these repetitive sequences, tandem and interspersed repeats were approximately 49.43 Mb (11.84% of the genome) and 212.22 Mb (50.81% of the genome), respectively. Additionally, retrotransposons and DNA transposons were primary components of the interspersed repeats accounting for 43.80 and 7.02% of the genome, respectively. The dominant type of retrotransposons was Long Terminal Retrotransposons (LTRs), which accounted for approximately 42.19% of the genome (35.00% LTR/Gypsy and 6.70% LTR/Copia retrotransposons) (Supplementary Table S5).

### Gene Prediction and Noncoding RNA Annotation

We applied multiple gene model prediction methods to accurately predict gene sets in the *S. yunnanensis* genome, including *de novo*, homology-based, and transcriptome-based methods. Analyses showed that a total of 25,467 protein-coding genes were predicted with an average gene length of 2,849.31 bp, an average coding DNA sequence (CDS) size of 1,194.81 bp, and average exons per gene of 5.24 (Table 1 and Supplementary Table S6). Moreover, 1,375 (96.1%) BUSCO genes were completely matched to our predicted *S. yunnanensis* gene sets, suggesting high completeness and accuracy of protein-coding genes (Supplementary Table S7). Overall, a total of 23,078 (90.62%) genes could be assigned to functional annotation within the public protein databases: TrEMBL (90.28%), Swiss-Prot (71.37%), NR (90.52%), EggNOG (42.55%), Gene Ontology (GO) (64.37%), Kyoto Encyclopedia of Genes and Genomes (KEGG) (25.24%), and InterPro (55.14%) (Supplementary Table S8). Additionally, noncoding RNAs were also identified, including 469 transfer RNAs (tRNAs), 617 ribosomal RNAs (rRNAs), 117 microRNAs (miRNAs), and 718 small nuclear RNAs (snRNAs) (Supplementary Table S9).

**TABLE 1:** Assembly and annotation summary of *Speranskia yunnanensis* genome.

Assembly feature	
Genome size (bp)	417,645,011
Longest contig (bp)	42,011,156
N50 of contig (bp)	12,521,520
GC ratio (%)	32.95
BUSCO score of assembly (%)	98.3
Number of genes	25,467
Percentage of repetitive sequence	69.11

### Gene Families and Phylogenetic Analysis

**FIGURE 1:**
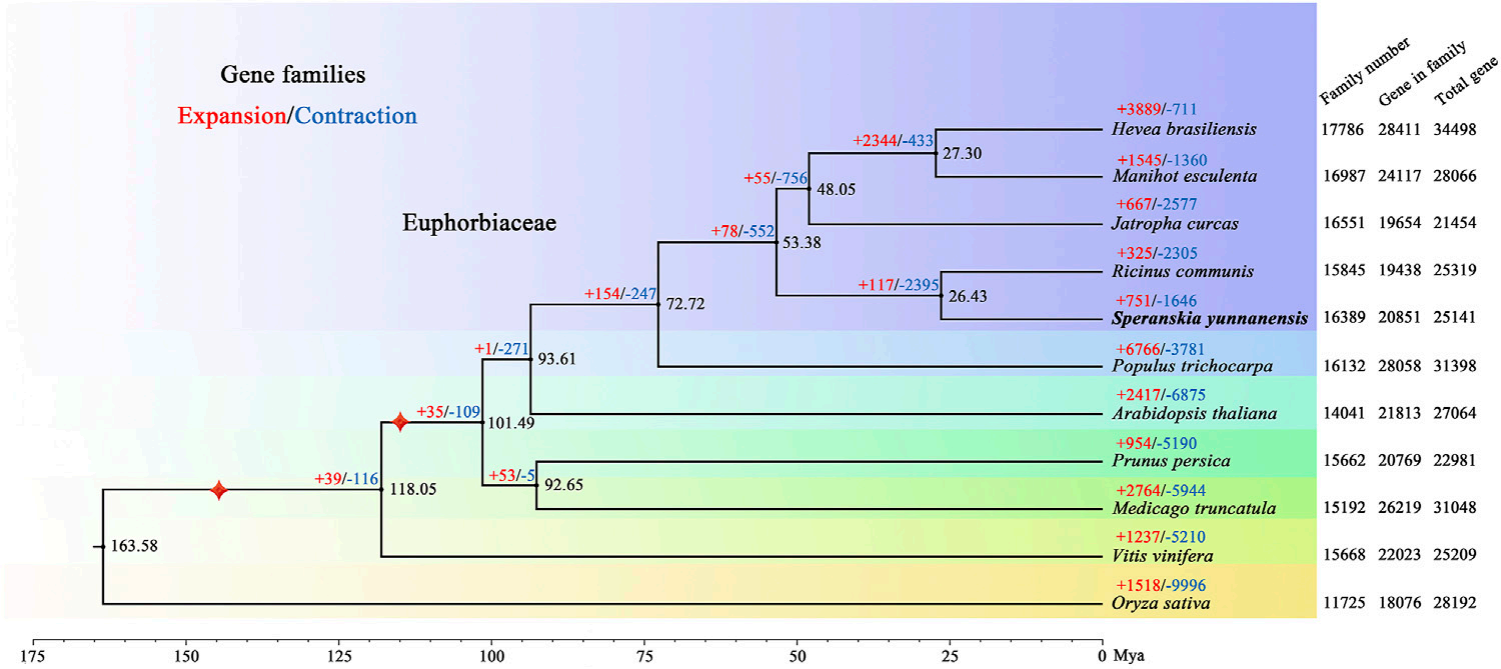
Phylogenetic tree of *S. yunnanensis* and 10 other species. Gene family expansions (+) and contractions (−) are indicated by red and blue, respectively. Black numbers represent divergence time between species. The numbers of gene families, clustered genes, and all predicted genes are indicated next to each species. Calibration point is marked by red star.

For phylogenetic analysis and discerning quantities of potential orthologous gene families, genes from 11 species, including *Arabidopsis thaliana, Hevea brasiliensis, Jatropha curcas, Manihot esculenta, Medicago truncatula, Oryza sativa, Prunus persica, Populus trichocarpa, Ricinus communis,* and *Vitis vinifera*, were clustered into gene families. In total, 20,851 *S. yunnanensis* genes (81.87%) were clustered into 16,389 gene families, of which 524 were unique to *S. yunnanensis* (Supplementary Table S10). Furthermore, *S. yunnanensis* shared 12,300 gene families with four other species (*H. brasiliensis, M. esculenta*, J. *curcas* and *R. communis*) and contained 635 unique gene families (Supplementary Figure S4). We identified and selected a total of 1,517 single-copy orthologous gene families for phylogenetic analyses and divergence time estimation. It was showed that *S. yunnanensis* and *R. communis* were closely related species in the Euphorbiaceae that diverged approximately 26.43 million years ago (Mya) (Figure 1 and Supplementary Figure S5).

We further discovered 751 expanded and 1,646 contracted gene families in *S. yunnanensis*. Among them, 85 gene families exhibited remarkable expansion and 30 gene families showed dramatic contraction (Supplementary Table S11). The enrichment results showed that the expanded genes were mainly related to GO terms in ion binding, such as zinc and iron (GO:0008270, GO:0046872, GO:0043169, GO:0005506, and GO:0043167), nuclease activity (GO:0004521, GO:0016891, and GO:0004540), the terpenoid synthesis process (GO:0046246 and GO:0016114) (Supplementary Table S12), and KEGG maps of metabolic synthesis of terpenoids (K09109, K00909, K00902, and K00904) (Supplementary Table S13). However, the contracted genes were mainly related to the ribosomal composition process (GO:0003735, GO:0022625, GO:0044391, GO:0015934, GO:0022626, GO:0005840, GO:0042788, and GO:0005844).

### Whole-Genome Duplication Analysis

To investigate polyploidization events within *S. yunnanensis*, we performed a comparative analysis of the genome sequences from *S. yunnanensis* and the other three species (*J. curcas, R. communis* and *V. vinifera*). By measuring the *Ks* rate of orthologous gene pairs, we found *S. yunnanensis* shared similar *Ks* distributions (with a peak *Ks* value of 1.52) with *J. curcas* and *R. communis*, indicating a shared, ancient polyploidy event (γ) of *S. yunnanensis* with *V. vinifera* whereas no additional, recent species-specific WGD event in the species (Supplementary Figure S6).

## Materials and Methods

### Sample Processing and Whole-Genome Sequencing

The natural plants of *S. yunnanensis* were collected from Zhenkang County, Yunnan Province, China. Genomic DNA was extracted from fresh young leaves using the QIAGEN Genomic reagent kit, according to the manufacturer’s protocols. On the basis of adequate quality sample, a paired-end library with short-insert sizes of 400 bp was prepared for sequencing on the HiSeq X Ten PE150 platform using standard Illumina instructions. Following the Nanopore library construction protocol, a Nanopore library was constructed and the long-read data was generated using the PromethION sequencer (Oxford Nanopore Technologies, United Kingdom). Sequencing adapters were removed, and reads of low quality and short length were filtered out. Freshly harvested leaves were used to construct the Hi-C libraries. First, the intranuclear chromatin was fixed with formaldehyde to facilitate covalent bond formation. Subsequently, MboI, a restriction endonuclease, was used to digest cross-linked DNA, after which sticky DNA ends were repaired with biotin-marked nucleotides and the resulting blunt ends were ligated together with DNA ligase. Proteins were then removed with proteases. To release DNA molecules from cross-links, purified DNA was randomly interrupted to fragments with an average size of 300 bp using ultrasound and attached to adapters. Biotin-labeled DNA fragments were ultimately captured and enriched using streptavidin beads to construct paired-end sequencing libraries, which were then sequenced on the Illumina HiSeq platform to obtain 2 × 150 bp Hi-C raw reads. Finally, to aid gene annotation, we performed RNA sequencing for fresh tissues of the leaf, root, seed and stem from the same plant using an Illumina HiSeq 2500 platform.

### Estimating of *S. yunnanensis* Genome Size

We first filtered Illumina reads using FASTP v0.20.0 (Chen et al., 2018) with default parameters. Clean reads were analyzed by KMC v3.1.1 (Marek et al., 2017) to generate the k-mer depth distribution with a k-mer size of 21 bp, and the final result was plotted in GenomeScope v2.0 (http://qb.cshl.edu/genomescope/genomescope2.0/) (Ranallo-Benavidez et al., 2020).

### Genome Assembly and Pseudochromosome Construction

High-quality controlled Oxford Nanopore Technologies long reads were capitalized on assembly using NextDenovo v2.4.0 (https://github.com/Nextomics/NextDenovo) with a read cutoff length of 4 kb. A preliminary assembly was achieved using NextGraph, a subprogram of NextDenovo. Then, iterative polishing was performed repetitively using NextPolish v1.3.1 (Hu et al., 2019). At this stage, Oxford Nanopore Technologies long reads and Illumina short-clean reads were used repeatedly for 10 rounds of genome correction after finalizing the preassembly. The completeness of genome assembly was assessed by BUSCO v3.0.2 (Simão et al., 2016) with the embryophyta_odb10 databases.

For Hi-C sequence data, we also initially filtered out low-quality reads using FASTP with default parameters. Clean Hi-C data were then analyzed using JUICER v1.6.2 (Durand et al., 2016) with default parameters, including softlinking and indexing the genome sequence in the references/folder, creating restriction enzyme proposed cutting sites, and initializing Hi-C reads. Subsequently, the resulting file was utilized as input into the 3D-DNA program (Dudchenko et al., 2017) with the parameters -r 2 -q 1 for further analysis. We used 3D-DNA to elaborately optimize the ordering and orientation of each clustered group, and scaffold the genome to produce a chromosomal level assembly. Finally, an interaction heatmap was corrected and plotted using JuiceBox v1.11.9 (Durand et al., 2016).

### Repeat Annotation

Repetitive elements, including tandem repeats and interspersed repeats, were predicted in the *S. yunnanensis* genome. Tandem Repeats Finder (TRF) v4.09 (Benson, 1999) was first applied to annotate tandem repeats. For interspersed repeats, both homology-based and *de novo* approaches were mainly used. RepeatMasker v4.0.7 (Tarailo-Graovac and Chen, 2009) and RepeatProteinMasker were used to identify interspersed repeats on the basis of homology alignment of the input *S. yunnanensis* genome sequence against Repbase v16.10 (Bao et al., 2015). RepeatModeler v5.8.8 (Smit and Hubley, 2008) was utilized to construct the repeat library, which comprised a repeat consensus database with classification information. Finally, RepeatMasker was employed to generate the *de novo* predictions.

### Gene Structure Annotation

Protein-coding annotations were predicted using a combination of *de novo*, homology-based, and transcriptome-based approaches based on the repeat masked genome. For *de novo* prediction, AUGUSTUS v3.2.3 (Stanke et al., 2008), GENSCAN (Burge and Karlin, 1998), and GlimmerHMM v3.0.4 (Majoros et al., 2004) were applied on the basis of the models trained with CDS data from *A. thaliana*. GeMoMa v1.7.1 (Keilwagen et al., 2016) was used for homology prediction, with protein sequences from *A. thaliana, H. brasiliensis*, *J. curcas*, *M. esculenta*, *P. persica*, *P. trichocarpa, R. communis*, and *V. vinifera*. For transcriptome-based prediction, after trimming low-quality bases and adapter sequences with Trimmomatic v0.39 (Bolger et al., 2014), nonredundant full-length transcriptomes from the *de novo* assembly using TRINITY v2.9.1 (Haas et al., 2013) were subsequently aligned to the genome to resolve gene structure using PASA v2.3.3 (Haas et al., 2003) with parameters -c RunPASA.config--TRANSDECODER -C -r -R --ALIGNERS blat, gmap. In addition, EvidenceModeler (EVM) v1.1.1 (Haas et al., 2008) was used to generate the final consensus set of the gene model using the previous three approaches. Ultimately, the completeness of the genome assembly was further assessed using BUSCO based on the embryophyta_odb10 databases.

### Gene Functional Annotation

Functional annotations of protein-coding genes were carried out using BLASTP (e-value 1e−5) v2.2.26 (Altschul et al., 1997) against publicly available databases including the Swiss-Prot (Bairoch and Apweiler, 2000), TrEMBL (Bairoch and Apweiler, 2000), NR (Pruitt et al., 2007) and eggNOG (Huerta-Cepas et al., 2015). Protein motifs and domains were annotated using InterProScan v5.30−69.0 (Jones et al., 2014) by searching ProDom, SMART, SUPERFAMILY and PRINTS. Potential pathways of each gene were found in the KEGG Automatic Annotation Server (https://www.genome.jp/kegg/kaas/) using the KEGG database.

### Noncoding RNA Annotation

Noncoding RNA genes, including tRNA, rRNA, miRNA, and snRNA, were predicted in the assembled genome. tRNA genes were predicted using tRNAscan-SE v1.3.1 (Lowe and Eddy, 1997) with eukaryote parameters, and rRNA with high conservation were predicted by aligning reads to the *Arabidopsis* template rRNA sequences using BLASTN (Altschul et al., 1997), with an e value of 1e−5. Additionally, INFERNAL (Nawrocki and Eddy, 2013) was used to predict miRNA and snRNA genes on the basis of the Rfam database (Griffiths-Jones et al., 2005).

### Gene Families and Phylogenetic Analysis

To reveal *S. yunnanensis* genome evolution, protein-coding gene sequences from 11 species were selected for phylogenetic analysis: *A. thaliana, H. brasiliensis, J. curcas, M. esculenta, M. truncatula, O. sativa, P. persica, P. trichocarpa, R. communis,* and *V. vinifera.* First, an all-vs-all BLASTP (e-value 1e−5) was applied to calculate gene similarities. And the paralogs and orthologs were respectively clustered using OrthoMCL v2.0.9 (Li et al., 2003). Second, single-copy orthologous genes were extracted from the OrthoMCL clustering results and aligned using MAFFT v7.453 (Katoh and Standley, 2013) with default parameters. The protein sequence alignments were converted into CDS alignments, which were then concatenated into a supergene for phylogenetic analysis. IQ-TREE v1.6.12 (Lam-Tung et al., 2015) was used to construct a maximum likelihood (ML) tree with the MFP model and a bootstrap of 1000. Subsequently, the ML tree was input into BaSeml v4.0 and MCMCTree v4.0 (Yang, 2007) to estimate the nucleotide substitution rates and the divergence times, respectively. The approximate divergence times between *A. thaliana* and *O. sativa* (115–308 Mya), as well as between *A. thaliana* and *V. vinifera* (107–135 Mya; http://www.timetree.org/) were used as calibrators in time estimation. The MCMCTree parameters were set as follows: model = 7, BDparas = 110, kappa_gamma = 62, alpha_gamma = 11, burnin = 500,000, sampfreq = 5,000, nsample = 20,000. Based on the divergence times estimated and the gene families identified via OrthoMCL, the expansion and contraction of gene families were predicted using CAFÉ v3.1 (Han et al., 2013) under a random birth-and-death model.

### Whole-Genome Duplication Analysis

Protein-coding sequences within one genome or between two different genomes were aligned using BLASTP with an e-value cutoff of 1e−5. Syntenic blocks and synonymous nucleotide substitutions (*Ks*) were determined from protein sequence alignments based on the detected homologous gene pairs using WGDI (https://github.com/SunPengChuan/wgdi). We further filtered the tandem duplicated gene pairs. WGD and speciation events were inferred from paralogous and orthologous pairs of *Ks* distribution peaks, respectively.

## Data Availability

Whole-genome sequence reads (including the Nanopore long reads, NGS short reads, and Hi-C reads) used in this study have been deposited in the NCBI database under the BioProject accession number PRJNA744706. The genome assembly file and genome annotation files (repeat annotation and gene structure annotation) are available at Figshare (https://figshare.com/articles/dataset/Speranskia_yunnanensis/15057930).
