# Supplementary material for: Chromosome-Level Genome Assembly of the Rare and Endangered Tropical Plant Speranskia yunnanensis (Euphorbiaceae)
Source: Front Genet. 2022 Jan 24;12:755564. doi: 10.3389/fgene.2021.755564 (PMC8819086; doi:10.3389/fgene.2021.755564)
Supplement: Supplementary file 1 [file DataSheet1.docx]

# Supplementary Figures


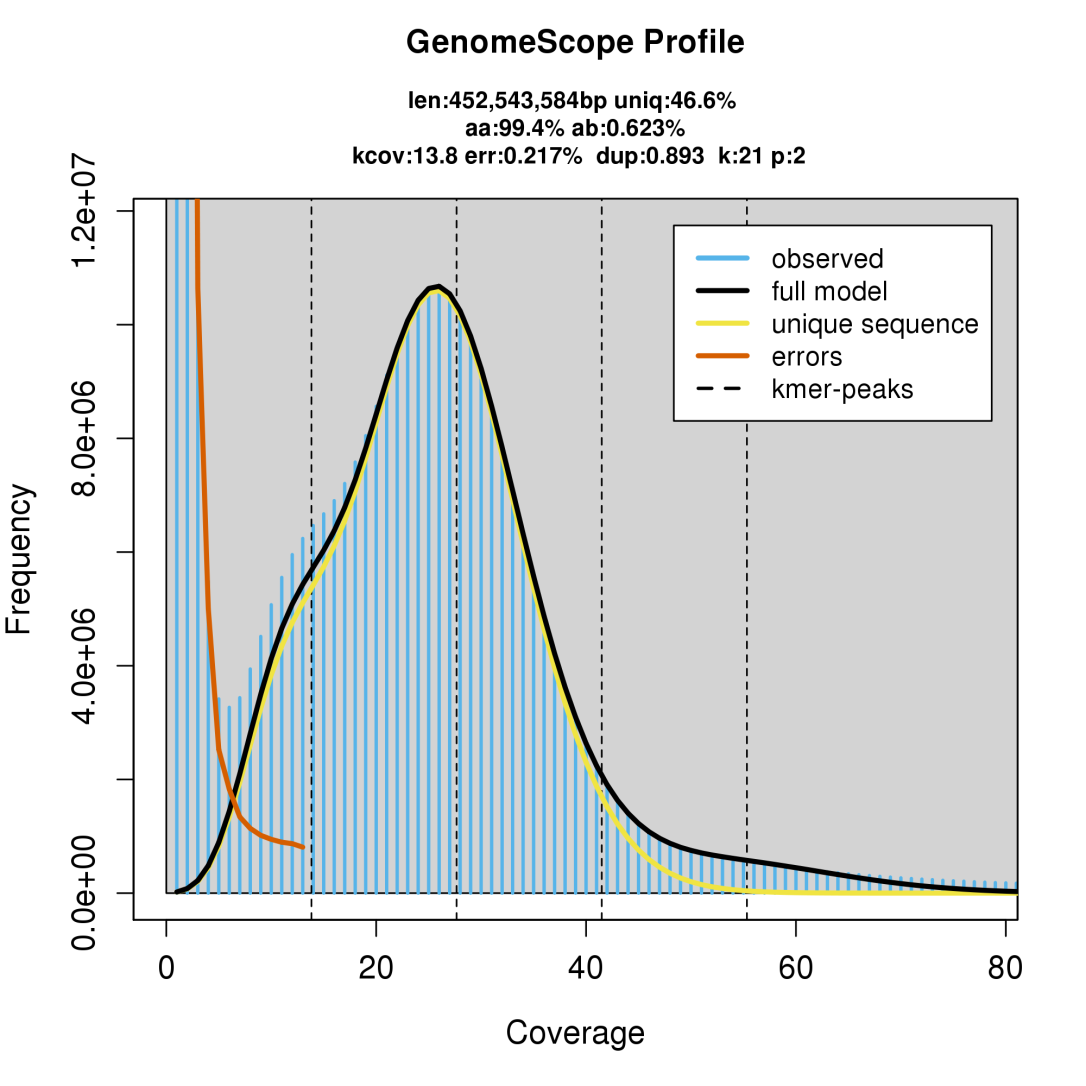


**Supplementary Figure 1.** Genome size and heterozygosity estimation of *S. yunnanensis* using 21 K-mer distribution.


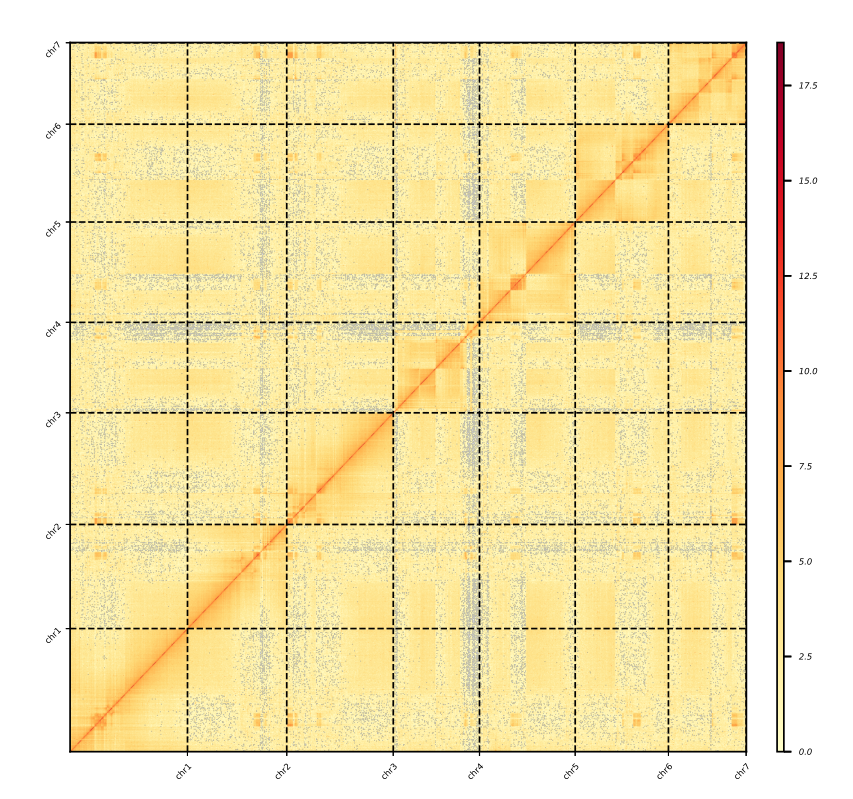


**Supplementary Figure 2.** Hi-C assisted assembly of *S. yunnanensis* pseudochromosomes. Heatmap showing Hi-C interactions.


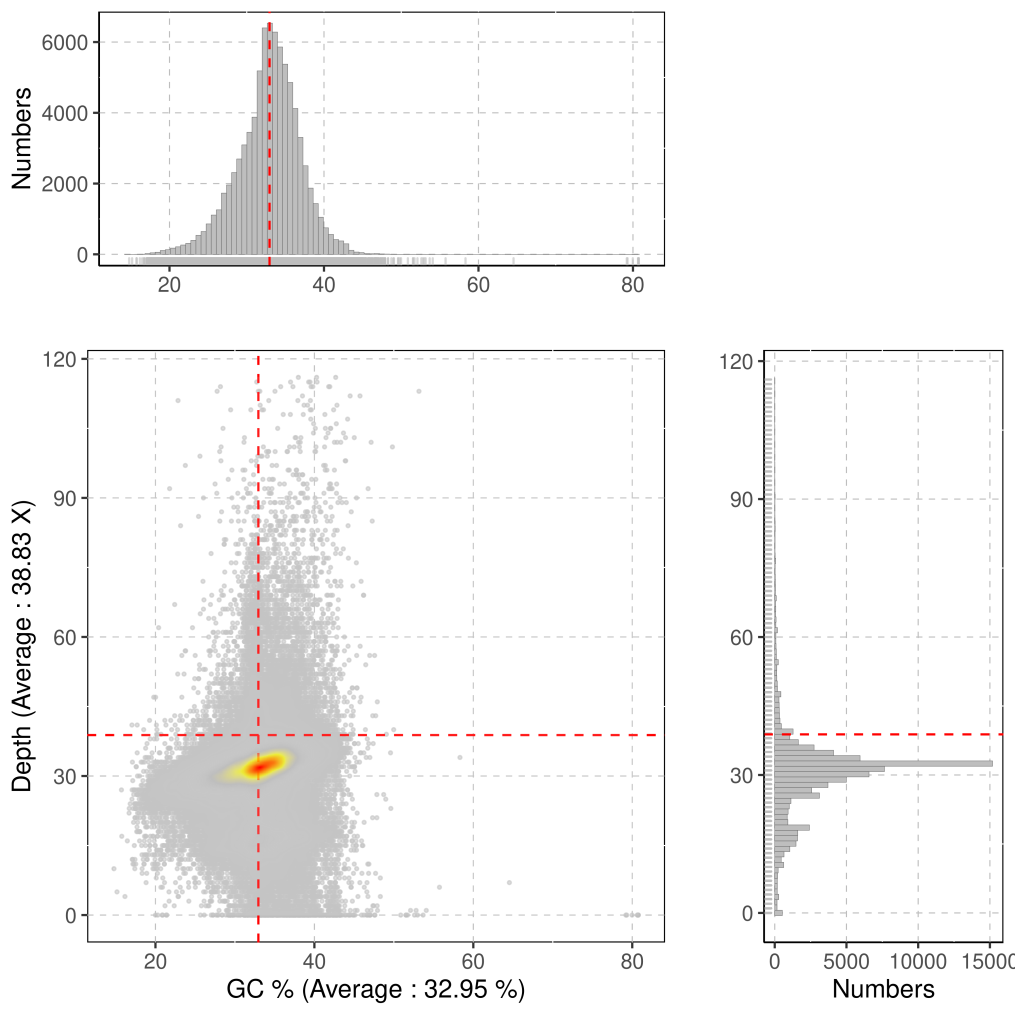


**Supplementary Figure 3. Distribution of genome GC content and sequencing depth.** The x-axis represents the GC content with 20kb windows, and the y-axis represents the sequencing depth with 30kb windows. The Poisson distribution proves the high quality of genome sequencing.





**Supplementary Figure 4.** The Venn diagram shows shared and unique gene families in *H. brasiliensis, J. curcas, M. esculenta*, *R. communis* and *S. yunnanensis*. The gene- family number is listed in each components.


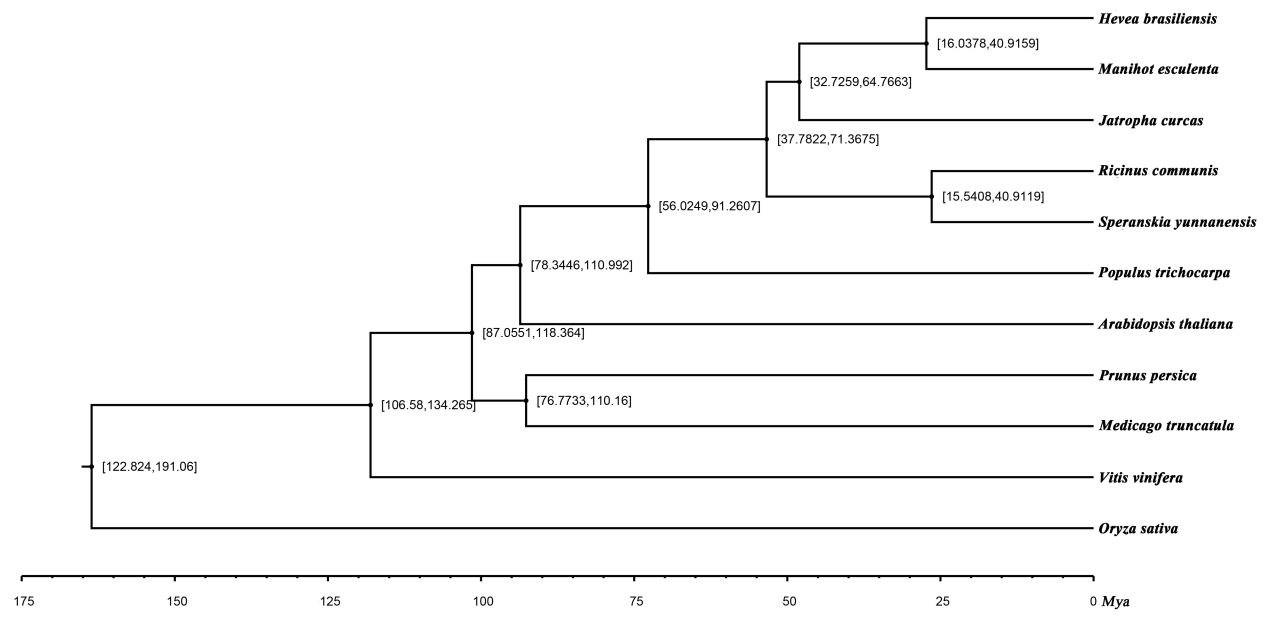


**Supplementary Figure 5.** The estimation of divergence times between species by MCMCtree program. The divergence time were estimated based on two reference divergence time (*A. thaliana* and *O. sativa*: 115~308 Mya; *A. thaliana* and *V. vinifera*: 107~135 Mya).

**
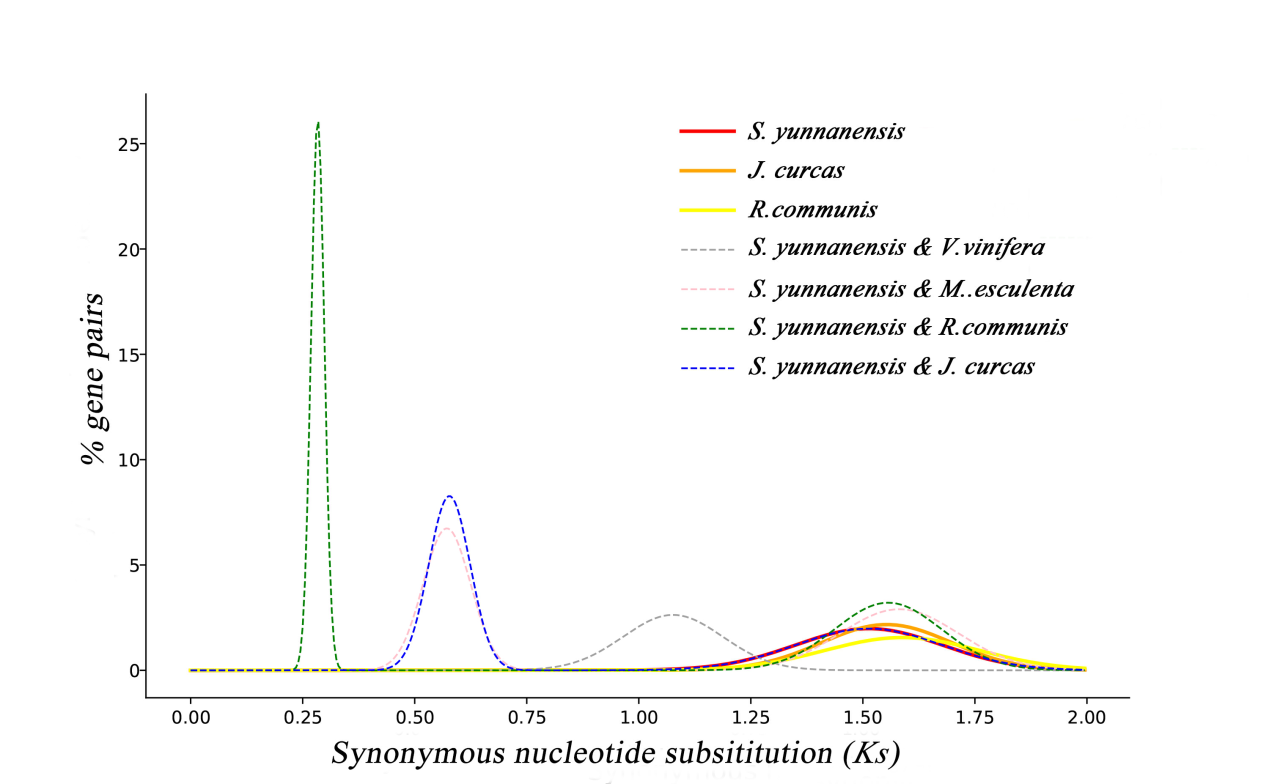
**

**Supplementary Figure 6.** Frequency distributions of synonymous nucleotide substitution (*Ks*) rates for homologous gene pairs. Solid and dotted lines represent paralogous and orthologous gene pairs, respectively.

**Supplementary Tables**

**Supplementary Table 1. Satistics for the assemblies of the *S. yunnanensis* genomes.**

| Stat Type | Preliminary Assembly | | Polish Genome | |
| --- | --- | --- | --- | --- |
|  | Contig Length(bp) | Contig Number | Contig Length(bp) | Contig Number |
| Total | 414,612,353 | 195 | 417,645,011 | 195 |
| Max | 41,687,520 | - | 42,011,156 | - |
| N50 | 12424576 | 9 | 12,521,520 | 9 |
| N60 | 9254715 | 13 | 9,319,929 | 13 |
| N70 | 5836130 | 19 | 5,876,254 | 19 |
| N80 | 3546015 | 27 | 3,578,131 | 27 |
| N90 | 903044 | 49 | 904,188 | 49 |

**Supplementary Table 2**. **The statistics of Hi-C assembly.**

| Chromosome | Size(Mb) | GC% | Gene |
| --- | --- | --- | --- |
| Chr1 | 68.49 | 31.13 | 4655 |
| Chr2 | 63.82 | 31.09 | 3937 |
| Chr3 | 62.89 | 31.18 | 3682 |
| Chr4 | 61.049 | 31.16 | 3771 |
| Chr5 | 54.519 | 31.06 | 3462 |
| Chr6 | 52.67 | 31.32 | 3072 |
| Chr7 | 45.31 | 31.40 | 2633 |
| Anchored | 408.62 | - |  |
| Unanchored | 9.02 | - | - |
| Anchored ratio (%) | 97.8 | - | - |
| Total | 417.64 | 31.03 | 25212 |

**Supplementary Table 3**. **Genome completeness measured by Benchmarking Universal SingleCopy Orthologs (BUSCO).**

| Type | Polish genome | |
| --- | --- | --- |
|  | Number | Percent(%) |
| Complete BUSCOs (C) | 1,352 | 98.3 |
| Complete and single-copy BUSCOs (S) | 1,326 | 96.4 |
| Complete and duplicated BUSCOs (D) | 26 | 1.9 |
| Fragmented BUSCOs (F) | 7 | 0.5 |
| Missing BUSCOs (M) | 16 | 1.2 |
| Total BUSCO groups searched | 1,375 | 100 |

**Supplementary Table 4. Assessment of genome consistency based on Illumina clean reads.**

| **Statistics** | **Value** |
| --- | --- |
| Clean Reads | 136419210 |
| Clean Bases (Mb) | 18383.95 |
| Mapped Reads | 128563844 |
| Mapping Rate (%) | 94.24% |
| Mean Depth (X) | 41.20 |
| Coverage Rate (%) | 95.12% |
| Coverage at least 4X (%) | 94.01% |
| Coverage at least 10X (%) | 91.33% |
| Coverage at least 30X (%) | 51.52% |

**Supplementary Table 5. Classification of repeats in the assembled genomes.**

| Repetitive element type | | Length (bp) of genome | Percentage (%) of Repeat | Percentage (%) of genome |
| --- | --- | --- | --- | --- |
| **SINE** | 9120 | | 0.003 | 0.002 |
| **LINE** | 6701492 | | 2.322 | 1.605 |
| L1 | 6502294 | | 2.253 | 1.557 |
| L2 | 11247 | | 0.004 | 0.003 |
| Other | 252422 | | 0.087 | 0.060 |
| **LTR** | 176203284 | | 61.044 | 42.190 |
| Copia | 27960836 | | 9.687 | 6.695 |
| Gypsy | 146160057 | | 50.636 | 34.996 |
| Other | 8165800 | | 2.829 | 1.955 |
| **DNA** | 29304308 | | 10.152 | 7.017 |
| CMC-EnSpm | 5227353 | | 1.811 | 1.252 |
| hAT-Ac | 5354506 | | 1.855 | 1.282 |
| hAT-Tip100 | 1643237 | | 0.569 | 0.393 |
| MuDR | 10925187 | | 3.785 | 2.616 |
| PIF-Harbinger | 1169999 | | 0.405 | 0.280 |
| Other | 5538704 | | 1.919 | 1.326 |
| **Transposable elements(TEs) total** | 212218204 | | 73.52 | 50.813 |
| **Satellites** | 585635 | | 0.203 | 0.140 |
| **Simple repeats** | 48843344 | | 16.921 | 11.695 |
| **Unclassified** | 84851475 | | 28.693 | 20.317 |
| **Total repeats** | 288648634 | | - | 69.11 |

**Supplementary Table 6.** **Prediction of protein-coding genes in *S. yunnanensis* genome.**

|  | Gene set | Total Genes Predicted | Average Gene Length (bp) | Average CDS Length (bp) | Average Exons per Gene | Average Exon Length (bp) | Average Intron Length (bp) |
| --- | --- | --- | --- | --- | --- | --- | --- |
| **De novo** | ***Augustus*** | 24841 | 3785.34 | 1210.83 | 5.46 | 221.94 | 342.97 |
|  | ***Genscan*** | 20783 | 10476.86 | 1320.49 | 6.29 | 209.79 | 1729.50 |
|  | ***Glimmerhmm*** | 25023 | 2261.26 | 957.82 | 4.24 | 225.87 | 402.23 |
| **Homolog** | ***P. persica*** | 21803 | 3213.08 | 1369.25 | 5.43 | 252.34 | 416.57 |
|  | ***A. thaliana*** | 19784 | 3213.64 | 1311.51 | 5.61 | 233.72 | 412.47 |
|  | ***H. brasiliensis*** | 24967 | 3068.20 | 1311.98 | 5.18 | 253.10 | 419.79 |
|  | ***J. curcas*** | 22922 | 3149.70 | 1340.58 | 5.37 | 249.57 | 413.83 |
|  | ***M. esculenta*** | 22610 | 3218.23 | 1361.44 | 5.44 | 250.12 | 417.91 |
|  | ***P. trichocarpa*** | 21199 | 3323.63 | 1386.61 | 5.61 | 246.92 | 419.67 |
|  | ***R. communis*** | 25456 | 3191.68 | 1181.64 | 5.12 | 231.83 | 486.08 |
|  | ***V.*** ***vinifera*** | 21907 | 3208.84 | 1353.41 | 5.39 | 251.27 | 423.02 |
| ***RNA-seq*** | | 48095 | 4726.68 | 1096.02 | 5.28 | 207.56 | 417.57 |
| **EVM** | | 25467 | 2849.31 | 1194.81 | 5.24 | 227.81 | 389.77 |

**Supplementary Table 7. Gene set completeness measured by Benchmarking Universal SingleCopy Orthologs (BUSCO).**

| **Type** | **Number** | **Percent (%)** |
| --- | --- | --- |
| Complete BUSCOs (C) | 1,322 | 96.1 |
| Complete and single-copy BUSCOs (S) | 1,298 | 94.4 |
| Complete and duplicated BUSCOs (D) | 24 | 1.7 |
| Fragmented BUSCOs (F) | 25 | 1.8 |
| Missing BUSCOs (M) | 28 | 2.1 |
| Total BUSCO groups searched | 1,375 | 100 |

**Supplementary Table 8. Functional annotation of the predicted genes** **in *S. yunnanensis*.**

|  | **Database** | **Gene number** | **Percent (%)** |
| --- | --- | --- | --- |
|  | InterPro | 14042 | 55.14 |
|  | EggNOG | 10837 | 42.55 |
|  | GO | 16392 | 64.37 |
|  | KEGG | 6428 | 25.24 |
|  | Swiss-Prot | 18175 | 71.37 |
|  | TrEMBL | 22992 | 90.28 |
|  | NR | 23052 | 90.52 |
| Annotated |  | 23078 | 90.62 |
| Total |  | 25467 | 100 |

**Supplementary Table 9. Prediction of noncoding RNAs in the *S. yunnanensis* genomes.**

| **Software** | **Type** | **Total length (bp)** | **Number** | **Average length(bp)** |
| --- | --- | --- | --- | --- |
| INFERNAL | miRNA | 14509 | 117 | 124.01 |
|  | snRNA | 77266 | 718 | 107.61 |
| tRNAscan-SE | tRNA | 35302 | 469 | 75.27 |
| BLASTN | rRNA | 90899 | 617 | 147.32 |

**Supplementary Table 10. Statistics of the clustered gene family for 11 plant species.**

| **Species** | **Total genes** | **Genes in families** | **Families** | **Unique families** |
| --- | --- | --- | --- | --- |
| *A. thaliana* | 27064 | 21813 | 14041 | 883 |
| *H. brasiliensis* | 34498 | 28411 | 17786 | 445 |
| *J. curcas* | 21454 | 19654 | 16551 | 185 |
| *M. esculenta* | 28066 | 24117 | 16987 | 194 |
| *M. truncatula* | 31048 | 26219 | 15192 | 939 |
| *O. sativa* | 28192 | 18076 | 11725 | 1161 |
| *P. persica* | 22981 | 20769 | 15662 | 383 |
| *P. trichocarpa* | 31398 | 28058 | 16132 | 390 |
| *R. communis* | 25319 | 19438 | 15845 | 421 |
| *S. yunnanensis* | 25141 | 20851 | 16389 | 524 |
| *V. vinifera* | 25209 | 22023 | 15668 | 459 |

**Supplementary Table 11. Statistics of the expanded and contracted gene family for 11 plant species.**

| **Species** | **Families** | **Expansive families** | **Significant expansive families** | **Contractive families** | **Significant contractive families** |
| --- | --- | --- | --- | --- | --- |
| *A. thaliana* | 14041 | 2417 | 50 | 6875 | 20 |
| *H. brasiliensis* | 17786 | 3889 | 106 | 711 | 10 |
| *J. curcas* | 16551 | 667 | 51 | 2577 | 3 |
| *M. esculenta* | 16987 | 1545 | 65 | 1360 | 15 |
| *M. truncatula* | 15192 | 2764 | 123 | 5944 | 2 |
| *O. sativa* | 11725 | 1518 | 16 | 9996 | 16 |
| *P. persica* | 15662 | 954 | 79 | 5190 | 5 |
| *P. trichocarpa* | 16132 | 6766 | 87 | 3781 | 0 |
| *R. communis* | 15845 | 325 | 29 | 2305 | 21 |
| *S. yunnanensis* | 16389 | 751 | 85 | 1646 | 30 |
| *V. vinifera* | 15668 | 1237 | 108 | 5210 | 2 |

**Supplementary Table 12. Gene ontology (GO) enrichment analysis of the expanded and contracted gene families of *S. yunnanensis*.**

| **^1^Type** | **GO ID** | **GO Term** | **Number of enriched genes** | **Number of genes in background** | **Adjusted P-value** |
| --- | --- | --- | --- | --- | --- |
| **Expansion** |  |  |  |  |  |
| MF | GO:0046914 | transition metal ion binding | 259 | 1366 | 1.46E-13 |
| MF | GO:0008270 | zinc ion binding | 173 | 786 | 2.19E-13 |
| MF | GO:0046872 | metal ion binding | 315 | 1943 | 6.32E-13 |
| MF | GO:0043169 | cation binding | 315 | 2003 | 2.35E-11 |
| MF | GO:0010333 | terpene synthase activity | 29 | 67 | 1.19E-09 |
| MF | GO:0003843 | 1,3-beta-D-glucan synthase activity | 11 | 11 | 2.42E-09 |
| MF | GO:0004523 | RNA-DNA hybrid ribonuclease activity | 24 | 52 | 1.02E-08 |
| MF | GO:0016838 | carbon-oxygen lyase activity, acting on phosphates | 29 | 74 | 1.39E-08 |
| MF | GO:0004521 | endoribonuclease activity | 34 | 103 | 7.59E-08 |
| MF | GO:0016891 | endoribonuclease activity, producing 5'-phosphomonoesters | 29 | 80 | 9.72E-08 |
| MF | GO:0016893 | endonuclease activity, active with either ribo- or deoxyribonucleic acids and producing 5'-phosphomonoesters | 29 | 84 | 3.25E-07 |
| MF | GO:0005506 | iron ion binding | 76 | 369 | 9.13E-07 |
| MF | GO:0016705 | oxidoreductase activity, acting on paired donors, with incorporation or reduction of molecular oxygen | 81 | 420 | 5.89E-06 |
| MF | GO:0043546 | molybdopterin cofactor binding | 8 | 9 | 6.85E-06 |
| MF | GO:0016835 | carbon-oxygen lyase activity | 38 | 150 | 1.52E-05 |
| MF | GO:0004540 | ribonuclease activity | 37 | 145 | 1.66E-05 |
| MF | GO:0043167 | ion binding | 527 | 4114 | 2.24E-05 |
| MF | GO:0018479 | benzaldehyde dehydrogenase (NAD+) activity | 6 | 6 | 5.67E-05 |
| MF | GO:0010293 | abscisic aldehyde oxidase activity | 6 | 6 | 5.67E-05 |
| MF | GO:0018488 | aryl-aldehyde oxidase activity | 6 | 6 | 5.67E-05 |
| MF | GO:0050302 | indole-3-acetaldehyde oxidase activity | 6 | 6 | 5.67E-05 |
| MF | GO:0016623 | oxidoreductase activity, acting on the aldehyde or oxo group of donors, oxygen as acceptor | 6 | 6 | 5.67E-05 |
| MF | GO:0019115 | benzaldehyde dehydrogenase [NAD(P)+] activity | 6 | 6 | 5.67E-05 |
| MF | GO:0004031 | aldehyde oxidase activity | 6 | 6 | 5.67E-05 |
| MF | GO:0140098 | catalytic activity, acting on RNA | 89 | 510 | 6.87E-05 |
| MF | GO:0035251 | UDP-glucosyltransferase activity | 37 | 158 | 9.51E-05 |
| MF | GO:0004497 | monooxygenase activity | 41 | 186 | 1.42E-04 |
| MF | GO:0004029 | aldehyde dehydrogenase (NAD+) activity | 9 | 17 | 4.88E-04 |
| MF | GO:0004030 | aldehyde dehydrogenase [NAD(P)+] activity | 9 | 17 | 4.88E-04 |
| MF | GO:0008395 | steroid hydroxylase activity | 8 | 14 | 6.47E-04 |
| MF | GO:0010334 | sesquiterpene synthase activity | 11 | 26 | 7.74E-04 |
| MF | GO:0016829 | lyase activity | 56 | 305 | 9.15E-04 |
| MF | GO:0015297 | antiporter activity | 31 | 138 | 0.001052 |
| MF | GO:0030597 | RNA glycosylase activity | 16 | 51 | 0.001079 |
| MF | GO:0030598 | rRNA N-glycosylase activity | 16 | 51 | 0.001079 |
| MF | GO:0000287 | magnesium ion binding | 37 | 180 | 0.001469 |
| MF | GO:0140102 | catalytic activity, acting on a rRNA | 19 | 69 | 0.00154 |
| MF | GO:0046527 | glucosyltransferase activity | 37 | 187 | 0.002861 |
| MF | GO:0004826 | phenylalanine-tRNA ligase activity | 5 | 7 | 0.003847 |
| MF | GO:0022804 | active transmembrane transporter activity | 70 | 429 | 0.003784 |
| MF | GO:0004252 | serine-type endopeptidase activity | 27 | 130 | 0.008669 |
| MF | GO:0050660 | flavin adenine dinucleotide binding | 28 | 138 | 0.009806 |
| MF | GO:0016709 | oxidoreductase activity, acting on paired donors, with incorporation or reduction of molecular oxygen, NAD(P)H as one donor, and incorporation of one atom of oxygen | 26 | 125 | 0.009937 |
| MF | GO:0020037 | heme binding | 68 | 434 | 0.012888 |
| MF | GO:0016758 | transferase activity, transferring hexosyl groups | 64 | 405 | 0.014109 |
| MF | GO:0016760 | cellulose synthase (UDP-forming) activity | 9 | 26 | 0.014006 |
| MF | GO:0005230 | extracellular ligand-gated ion channel activity | 7 | 17 | 0.015413 |
| MF | GO:0022824 | transmitter-gated ion channel activity | 7 | 17 | 0.015413 |
| MF | GO:0022835 | transmitter-gated channel activity | 7 | 17 | 0.015413 |
| MF | GO:0004970 | ionotropic glutamate receptor activity | 7 | 17 | 0.015413 |
| MF | GO:0030594 | neurotransmitter receptor activity | 7 | 17 | 0.015413 |
| MF | GO:0016875 | ligase activity, forming carbon-oxygen bonds | 17 | 72 | 0.015898 |
| MF | GO:0004812 | aminoacyl-tRNA ligase activity | 17 | 72 | 0.015898 |
| MF | GO:0016759 | cellulose synthase activity | 9 | 27 | 0.016443 |
| MF | GO:0046906 | tetrapyrrole binding | 70 | 457 | 0.016498 |
| MF | GO:0071949 | FAD binding | 16 | 67 | 0.01789 |
| MF | GO:0008066 | glutamate receptor activity | 7 | 18 | 0.018315 |
| MF | GO:0003777 | microtubule motor activity | 17 | 75 | 0.022018 |
| MF | GO:0034007 | S-linalool synthase activity | 5 | 10 | 0.022412 |
| MF | GO:0016491 | oxidoreductase activity | 195 | 1500 | 0.022787 |
| MF | GO:0043531 | ADP binding | 18 | 83 | 0.026668 |
| MF | GO:0015399 | primary active transmembrane transporter activity | 34 | 195 | 0.02983 |
| MF | GO:0016799 | hydrolase activity, hydrolyzing N-glycosyl compounds | 16 | 72 | 0.03315 |
| MF | GO:0033897 | ribonuclease T2 activity | 5 | 11 | 0.034383 |
| MF | GO:0080013 | (E,E)-geranyllinalool synthase activity | 5 | 11 | 0.034383 |
| MF | GO:0045735 | nutrient reservoir activity | 9 | 32 | 0.041169 |
| MF | GO:0008194 | UDP-glycosyltransferase activity | 39 | 239 | 0.041172 |
| MF | GO:0016892 | endoribonuclease activity, producing 3'-phosphomonoesters | 5 | 12 | 0.044592 |
| CC | GO:0000148 | 1,3-beta-D-glucan synthase complex | 11 | 11 | 9.34E-11 |
| CC | GO:0098797 | plasma membrane protein complex | 11 | 33 | 0.002103 |
| CC | GO:0005871 | kinesin complex | 17 | 73 | 0.00145 |
| CC | GO:0017101 | aminoacyl-tRNA synthetase multienzyme complex | 7 | 13 | 0.001112 |
| CC | GO:0016592 | mediator complex | 11 | 44 | 0.008996 |
| CC | GO:0090406 | pollen tube | 13 | 60 | 0.010306 |
| CC | GO:0048046 | apoplast | 36 | 281 | 0.012098 |
| CC | GO:0005875 | microtubule associated complex | 17 | 100 | 0.017831 |
| CC | GO:0090404 | pollen tube tip | 5 | 12 | 0.023198 |
| CC | GO:0000314 | organellar small ribosomal subunit | 5 | 12 | 0.023198 |
| CC | GO:0098807 | chloroplast thylakoid membrane protein complex | 6 | 18 | 0.024958 |
| CC | GO:0010287 | plastoglobule | 12 | 65 | 0.032911 |
| BP | GO:0019748 | secondary metabolic process | 76 | 337 | 4.17E-11 |
| BP | GO:0044550 | secondary metabolite biosynthetic process | 55 | 227 | 4.80E-09 |
| BP | GO:0055114 | oxidation-reduction process | 203 | 1444 | 5.81E-09 |
| BP | GO:0006075 | (1->3)-beta-D-glucan biosynthetic process | 11 | 12 | 1.74E-08 |
| BP | GO:0006855 | drug transmembrane transport | 34 | 115 | 8.85E-08 |
| BP | GO:0015893 | drug transport | 34 | 117 | 1.23E-07 |
| BP | GO:0046246 | terpene biosynthetic process | 19 | 41 | 1.22E-07 |
| BP | GO:0042214 | terpene metabolic process | 19 | 44 | 4.73E-07 |
| BP | GO:0019759 | glycosinolate catabolic process | 10 | 13 | 1.76E-06 |
| BP | GO:0019762 | glucosinolate catabolic process | 10 | 13 | 1.76E-06 |
| BP | GO:0016145 | S-glycoside catabolic process | 10 | 13 | 1.76E-06 |
| BP | GO:0051274 | beta-glucan biosynthetic process | 20 | 52 | 1.35E-06 |
| BP | GO:0071417 | cellular response to organonitrogen compound | 18 | 43 | 1.43E-06 |
| BP | GO:0006074 | (1->3)-beta-D-glucan metabolic process | 11 | 18 | 7.94E-06 |
| BP | GO:0051273 | beta-glucan metabolic process | 20 | 61 | 2.43E-05 |
| BP | GO:0019757 | glycosinolate metabolic process | 24 | 87 | 5.35E-05 |
| BP | GO:0019760 | glucosinolate metabolic process | 24 | 87 | 5.35E-05 |
| BP | GO:0016143 | S-glycoside metabolic process | 24 | 87 | 5.35E-05 |
| BP | GO:1900457 | regulation of brassinosteroid mediated signaling pathway | 12 | 25 | 5.08E-05 |
| BP | GO:0006013 | mannose metabolic process | 7 | 9 | 1.55E-04 |
| BP | GO:0002218 | activation of innate immune response | 17 | 53 | 2.07E-04 |
| BP | GO:0120251 | hydrocarbon biosynthetic process | 19 | 65 | 2.43E-04 |
| BP | GO:1901699 | cellular response to nitrogen compound | 30 | 137 | 3.07E-04 |
| BP | GO:0051761 | sesquiterpene metabolic process | 11 | 25 | 3.60E-04 |
| BP | GO:0051762 | sesquiterpene biosynthetic process | 11 | 25 | 3.60E-04 |
| BP | GO:1900459 | positive regulation of brassinosteroid mediated signaling pathway | 7 | 10 | 3.66E-04 |
| BP | GO:1901658 | glycosyl compound catabolic process | 10 | 21 | 3.69E-04 |
| BP | GO:0071230 | cellular response to amino acid stimulus | 10 | 22 | 6.01E-04 |
| BP | GO:0002253 | activation of immune response | 17 | 58 | 5.96E-04 |
| BP | GO:0002758 | innate immune response-activating signal transduction | 13 | 37 | 7.61E-04 |
| BP | GO:0009850 | auxin metabolic process | 17 | 60 | 9.20E-04 |
| BP | GO:0016106 | sesquiterpenoid biosynthetic process | 13 | 38 | 9.96E-04 |
| BP | GO:0044273 | sulfur compound catabolic process | 10 | 25 | 0.002006 |
| BP | GO:0008299 | isoprenoid biosynthetic process | 36 | 198 | 0.001969 |
| BP | GO:0120252 | hydrocarbon metabolic process | 19 | 78 | 0.002699 |
| BP | GO:0002757 | immune response-activating signal transduction | 13 | 42 | 0.002971 |
| BP | GO:1901657 | glycosyl compound metabolic process | 35 | 195 | 0.003063 |
| BP | GO:0016114 | terpenoid biosynthetic process | 30 | 157 | 0.003065 |
| BP | GO:0006418 | tRNA aminoacylation for protein translation | 17 | 67 | 0.003488 |
| BP | GO:0002764 | immune response-regulating signaling pathway | 13 | 43 | 0.003521 |
| BP | GO:0071383 | cellular response to steroid hormone stimulus | 16 | 61 | 0.003436 |
| BP | GO:0043401 | steroid hormone mediated signaling pathway | 16 | 61 | 0.003436 |
| BP | GO:0009741 | response to brassinosteroid | 22 | 101 | 0.003484 |
| BP | GO:0071407 | cellular response to organic cyclic compound | 35 | 198 | 0.00348 |
| BP | GO:0044003 | modulation by symbiont of host process | 12 | 38 | 0.003638 |
| BP | GO:0048545 | response to steroid hormone | 16 | 62 | 0.003798 |
| BP | GO:0043038 | amino acid activation | 17 | 69 | 0.004223 |
| BP | GO:0043039 | tRNA aminoacylation | 17 | 69 | 0.004223 |
| BP | GO:0006432 | phenylalanyl-tRNA aminoacylation | 5 | 7 | 0.004572 |
| BP | GO:0014070 | response to organic cyclic compound | 58 | 394 | 0.004492 |
| BP | GO:0009616 | RNAi-mediated antiviral immune response | 8 | 19 | 0.005307 |
| BP | GO:0052018 | modulation by symbiont of RNA levels in host | 8 | 19 | 0.005307 |
| BP | GO:0052249 | modulation of RNA levels in other organism involved in symbiotic interaction | 8 | 19 | 0.005307 |
| BP | GO:0045089 | positive regulation of innate immune response | 17 | 71 | 0.005391 |
| BP | GO:0009250 | glucan biosynthetic process | 20 | 92 | 0.005935 |
| BP | GO:0009851 | auxin biosynthetic process | 11 | 35 | 0.006125 |
| BP | GO:0019722 | calcium-mediated signaling | 14 | 53 | 0.006489 |
| BP | GO:0051817 | modulation of process of other organism involved in symbiotic interaction | 12 | 41 | 0.006429 |
| BP | GO:0006595 | polyamine metabolic process | 8 | 20 | 0.007066 |
| BP | GO:0009688 | abscisic acid biosynthetic process | 9 | 25 | 0.007121 |
| BP | GO:1902645 | tertiary alcohol biosynthetic process | 9 | 25 | 0.007121 |
| BP | GO:0043289 | apocarotenoid biosynthetic process | 9 | 25 | 0.007121 |
| BP | GO:0009060 | aerobic respiration | 14 | 54 | 0.007293 |
| BP | GO:0009742 | brassinosteroid mediated signaling pathway | 14 | 54 | 0.007293 |
| BP | GO:0000463 | maturation of LSU-rRNA from tricistronic rRNA transcript (SSU-rRNA, 5.8S rRNA, LSU-rRNA) | 7 | 16 | 0.008468 |
| BP | GO:2000146 | negative regulation of cell motility | 5 | 8 | 0.008425 |
| BP | GO:0030336 | negative regulation of cell migration | 5 | 8 | 0.008425 |
| BP | GO:0051271 | negative regulation of CC movement | 5 | 8 | 0.008425 |
| BP | GO:0051701 | BP involved in interaction with host | 14 | 55 | 0.008218 |
| BP | GO:0017148 | negative regulation of translation | 20 | 96 | 0.008585 |
| BP | GO:0009699 | phenylpropanoid biosynthetic process | 18 | 82 | 0.008565 |
| BP | GO:0070301 | cellular response to hydrogen peroxide | 8 | 21 | 0.00865 |
| BP | GO:0046113 | nucleobase catabolic process | 6 | 12 | 0.0088 |
| BP | GO:0042343 | indole glucosinolate metabolic process | 6 | 12 | 0.0088 |
| BP | GO:0031589 | cell-substrate adhesion | 6 | 12 | 0.0088 |
| BP | GO:0034249 | negative regulation of cellular amide metabolic process | 20 | 97 | 0.008591 |
| BP | GO:0071367 | cellular response to brassinosteroid stimulus | 14 | 56 | 0.008491 |
| BP | GO:0055085 | transmembrane transport | 132 | 1117 | 0.011388 |
| BP | GO:0007018 | microtubule-based movement | 19 | 92 | 0.011344 |
| BP | GO:0043434 | response to peptide hormone | 5 | 9 | 0.013677 |
| BP | GO:0040013 | negative regulation of locomotion | 5 | 9 | 0.013677 |
| BP | GO:0043200 | response to amino acid | 10 | 34 | 0.014302 |
| BP | GO:0043692 | monoterpene metabolic process | 7 | 18 | 0.014884 |
| BP | GO:0043693 | monoterpene biosynthetic process | 7 | 18 | 0.014884 |
| BP | GO:0050778 | positive regulation of immune response | 17 | 80 | 0.014784 |
| BP | GO:0009932 | cell tip growth | 23 | 128 | 0.019772 |
| BP | GO:0019932 | second-messenger-mediated signaling | 14 | 62 | 0.020249 |
| BP | GO:1901652 | response to peptide | 5 | 10 | 0.020559 |
| BP | GO:0001824 | blastocyst development | 5 | 10 | 0.020559 |
| BP | GO:0080027 | response to herbivore | 5 | 10 | 0.020559 |
| BP | GO:0002684 | positive regulation of immune system process | 17 | 84 | 0.022088 |
| BP | GO:0016032 | viral process | 11 | 43 | 0.022476 |
| BP | GO:0009860 | pollen tube growth | 20 | 107 | 0.023587 |
| BP | GO:0010048 | vernalization response | 9 | 31 | 0.023409 |
| BP | GO:0006596 | polyamine biosynthetic process | 6 | 15 | 0.024668 |
| BP | GO:0006720 | isoprenoid metabolic process | 36 | 240 | 0.028071 |
| BP | GO:0042430 | indole-containing compound metabolic process | 15 | 72 | 0.029687 |
| BP | GO:0006714 | sesquiterpenoid metabolic process | 13 | 58 | 0.029602 |
| BP | GO:0002833 | positive regulation of response to biotic stimulus | 17 | 87 | 0.030863 |
| BP | GO:0042436 | indole-containing compound catabolic process | 5 | 11 | 0.03137 |
| BP | GO:0001701 | in utero embryonic development | 5 | 11 | 0.03137 |
| BP | GO:0009833 | plant-type primary cell wall biogenesis | 7 | 22 | 0.040015 |
| BP | GO:0045088 | regulation of innate immune response | 20 | 113 | 0.039912 |
| BP | GO:0031349 | positive regulation of defense response | 17 | 90 | 0.040344 |
| BP | GO:0032103 | positive regulation of response to external stimulus | 17 | 90 | 0.040344 |
| BP | GO:0098586 | cellular response to virus | 8 | 28 | 0.041172 |
| BP | GO:0006207 | 'de novo' pyrimidine nucleobase biosynthetic process | 5 | 12 | 0.043885 |
| BP | GO:1900458 | negative regulation of brassinosteroid mediated signaling pathway | 5 | 12 | 0.043885 |
| BP | GO:0045333 | cellular respiration | 16 | 84 | 0.041921 |
| BP | GO:0006928 | movement of cell or subCC | 26 | 164 | 0.041999 |
| BP | GO:0070588 | calcium ion transmembrane transport | 13 | 62 | 0.041733 |
| **Decrease** | |  |  |  |  |
| MF | GO:0003735 | structural constituent of ribosome | 15 | 272 | 8.73E-05 |
| MF | GO:0005198 | structural molecule activity | 16 | 357 | 0.000274 |
| CC | GO:0022625 | cytosolic large ribosomal subunit | 9 | 68 | 5.34E-06 |
| CC | GO:0044391 | ribosomal subunit | 12 | 157 | 6.59E-06 |
| CC | GO:0015934 | large ribosomal subunit | 9 | 89 | 1.92E-05 |
| CC | GO:0044445 | obsolete cytosolic part | 12 | 182 | 1.66E-05 |
| CC | GO:0022626 | cytosolic ribosome | 11 | 154 | 1.89E-05 |
| CC | GO:0005840 | ribosome | 15 | 319 | 2.85E-05 |
| CC | GO:0042788 | polysomal ribosome | 6 | 56 | 0.000601 |
| CC | GO:0005844 | polysome | 6 | 70 | 0.00189 |
| CC | GO:0071944 | cell periphery | 39 | 2380 | 0.021503 |
| CC | GO:0043228 | non-membrane-bounded organelle | 26 | 1361 | 0.020794 |
| CC | GO:0043232 | intracellular non-membrane-bounded organelle | 26 | 1361 | 0.020794 |
| CC | GO:0005886 | plasma membrane | 32 | 1933 | 0.040974 |
| BP | GO:0043043 | peptide biosynthetic process | 18 | 572 | 0.037456 |
| BP | GO:0043603 | cellular amide metabolic process | 21 | 738 | 0.025543 |
| BP | GO:0006412 | translation | 17 | 560 | 0.046585 |

^1^MF: molecular function; CC: cellular component; BP: biological process.

**Supplementary Table 13. KEGG enrichment analysis of the expanded and contracted gene families of *S. yunnanensis.***

| **Term Name** | **Number of genes in background** | **P-value** | **Adjusted P-value** |
| --- | --- | --- | --- |
| **Expansion** |  |  |  |
| B 09109 Metabolism of terpenoids and polyketides | 73 | 314 | 1.59E-13 |
| 00909 Sesquiterpenoid and triterpenoid biosynthesis | 19 | 51 | 1.12E-06 |
| 00902 Monoterpenoid biosynthesis | 17 | 42 | 1.01E-06 |
| 00480 Glutathione metabolism | 27 | 108 | 9.16E-06 |
| B 09106 Metabolism of other amino acids | 44 | 261 | 2.46E-04 |
| 00380 Tryptophan metabolism | 20 | 87 | 8.67E-04 |
| 00199 Cytochrome P450 | 23 | 112 | 0.001327 |
| 00905 Brassinosteroid biosynthesis | 8 | 21 | 0.004109 |
| 04120 Ubiquitin mediated proteolysis | 29 | 176 | 0.007415 |
| 00904 Diterpenoid biosynthesis | 13 | 56 | 0.01123 |
| A09100 Metabolism | 287 | 2938 | 0.012441 |
| 00906 Carotenoid biosynthesis | 10 | 43 | 0.037499 |
| A09150 Organismal Systems | 39 | 295 | 0.047373 |
| B 09159 Environmental adaptation | 39 | 295 | 0.047373 |
| 00330 Arginine and proline metabolism | 13 | 68 | 0.047952 |
| 04626 Plant-pathogen interaction | 34 | 251 | 0.046193 |
| 01007 Amino acid related enzymes | 19 | 117 | 0.044063 |
| 00903 Limonene and pinene degradation | 5 | 14 | 0.041882 |
| **Decrese** |  |  |  |
| 03010 Ribosome | 16 | 291 | 0.00058 |
| 03011 Ribosome | 16 | 291 | 0.00058 |
| 04147 Exosome | 17 | 494 | 0.033808 |
